# Supplementary material for: Chemical Language Model Linker: blending text and molecules with modular adapters
Source: ArXiv. 2025 Apr 16:arXiv:2410.20182v2. Preprint. [Version 2] (PMC12047907)
Supplement: Supplementary file 1 [file NIHPP2410.20182v2-supplement-1.pdf]

Supplementary Figures

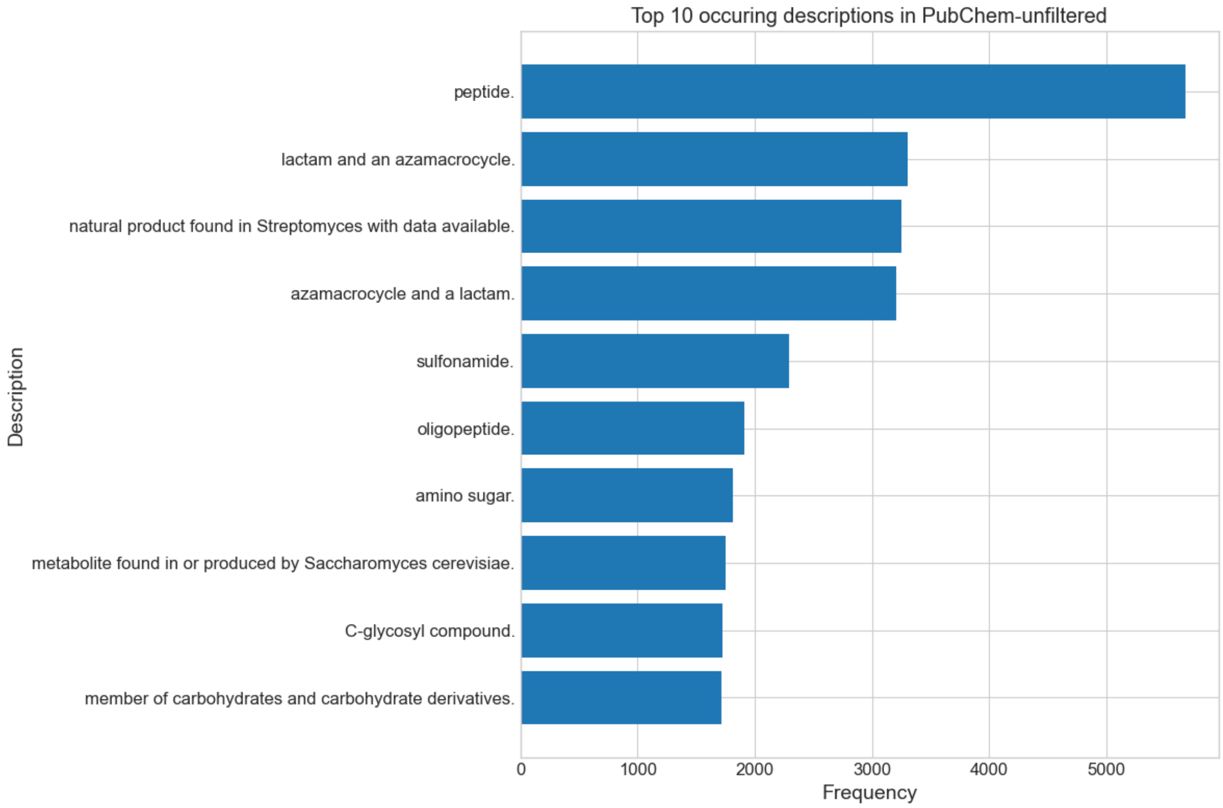

Figure S1: Top 10 most frequent descriptions from the PubChem-unfiltered dataset. The description prefix "The molecule is a/an" is omitted.

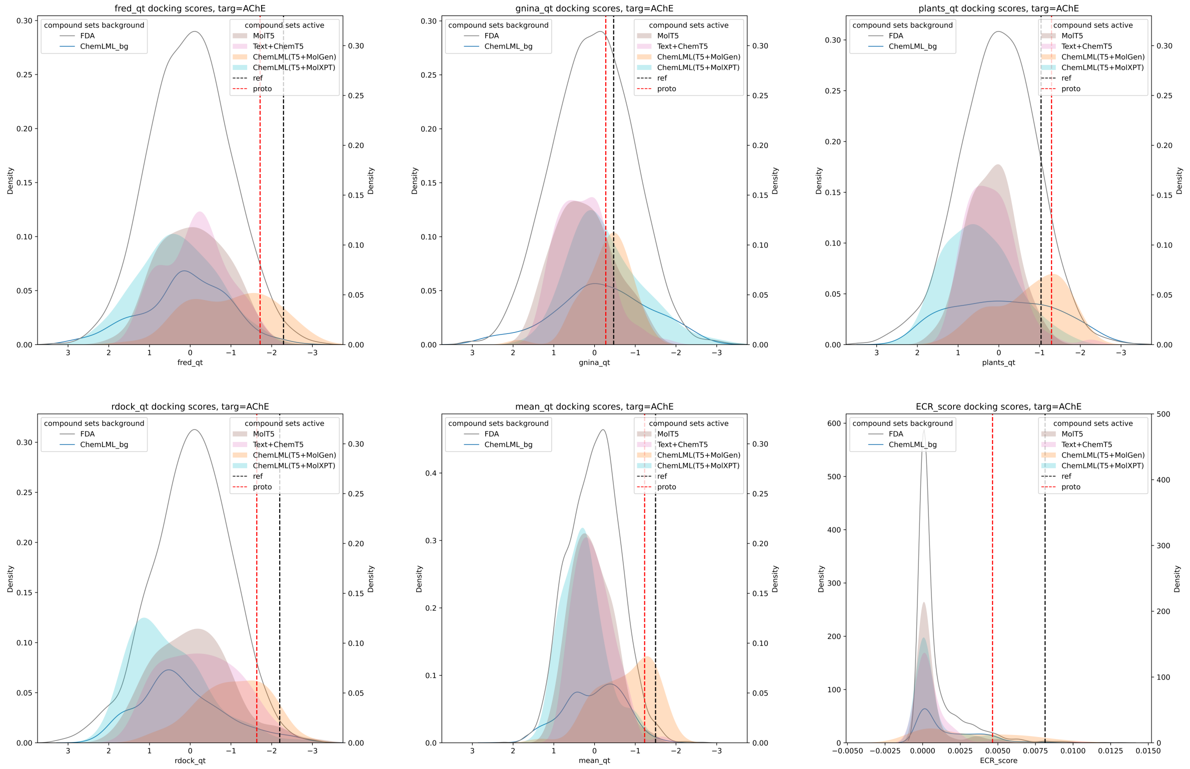

Figure S2: Individual docking program scores for AChE.

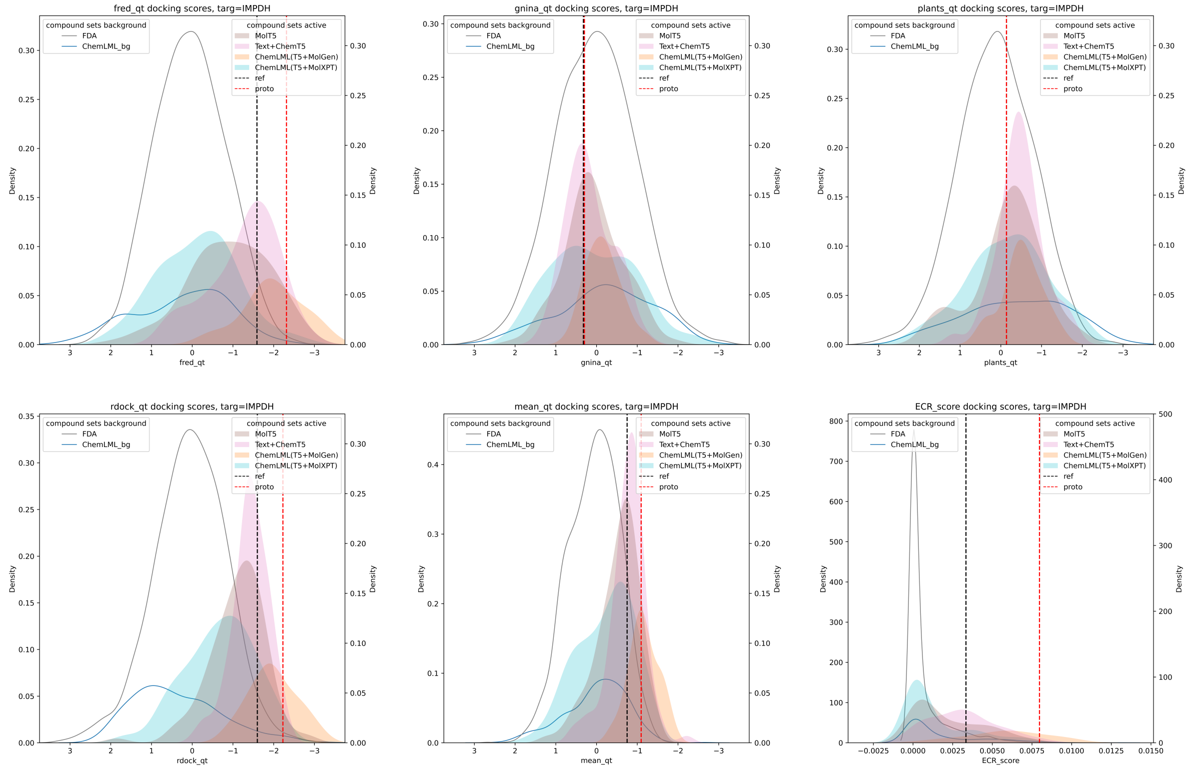

Figure S3: Individual docking program scores for IMPDH.

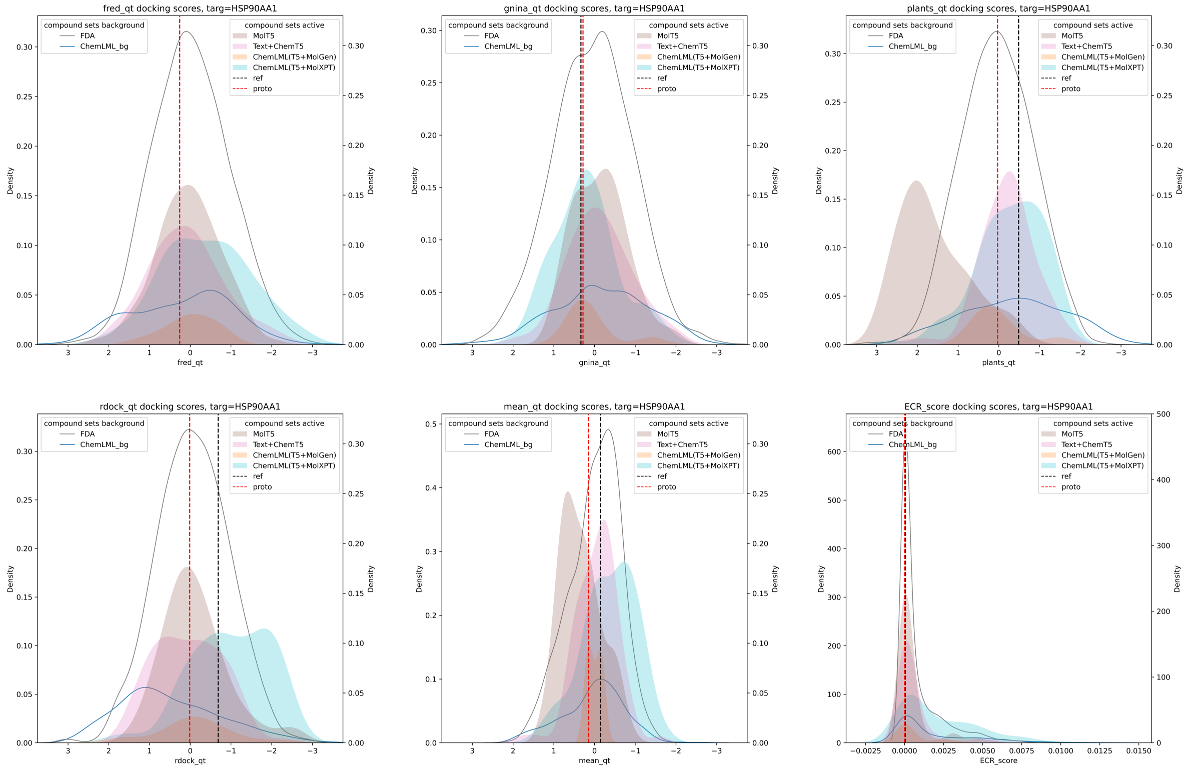

Figure S4: Individual docking program scores for HSP90AA1.

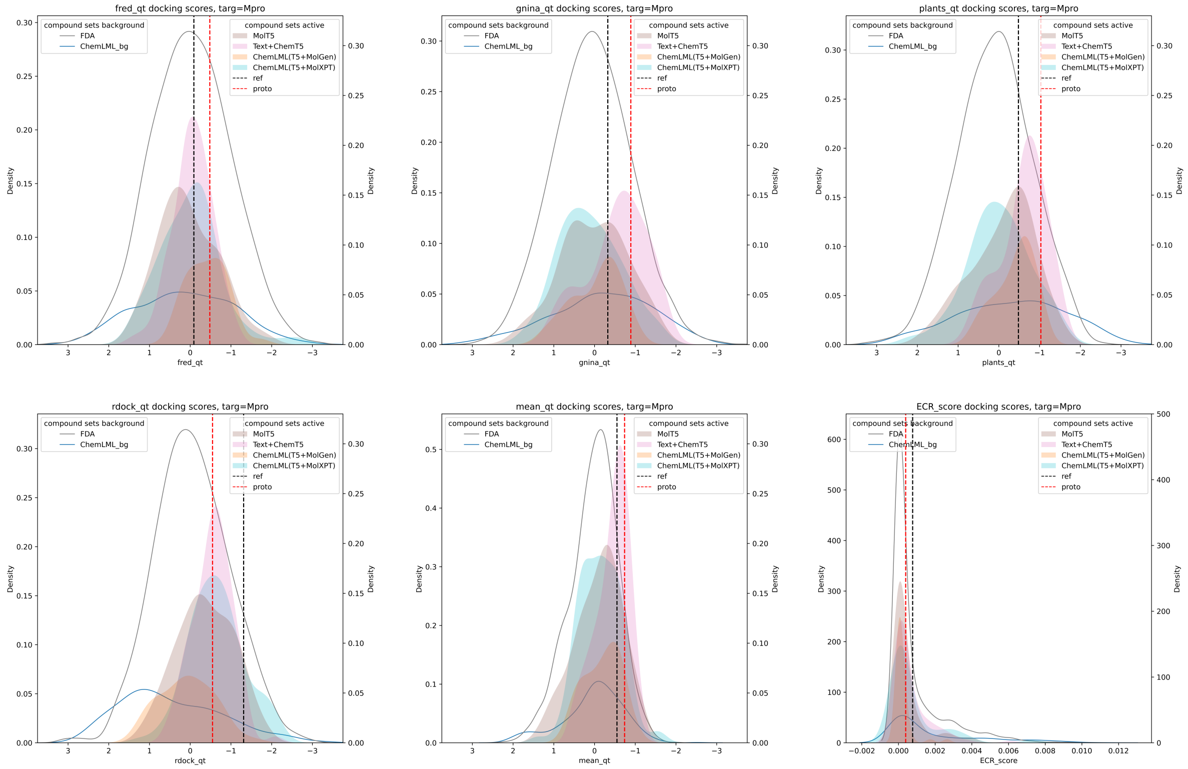

Figure S5: Individual docking program scores for M^{pro}.

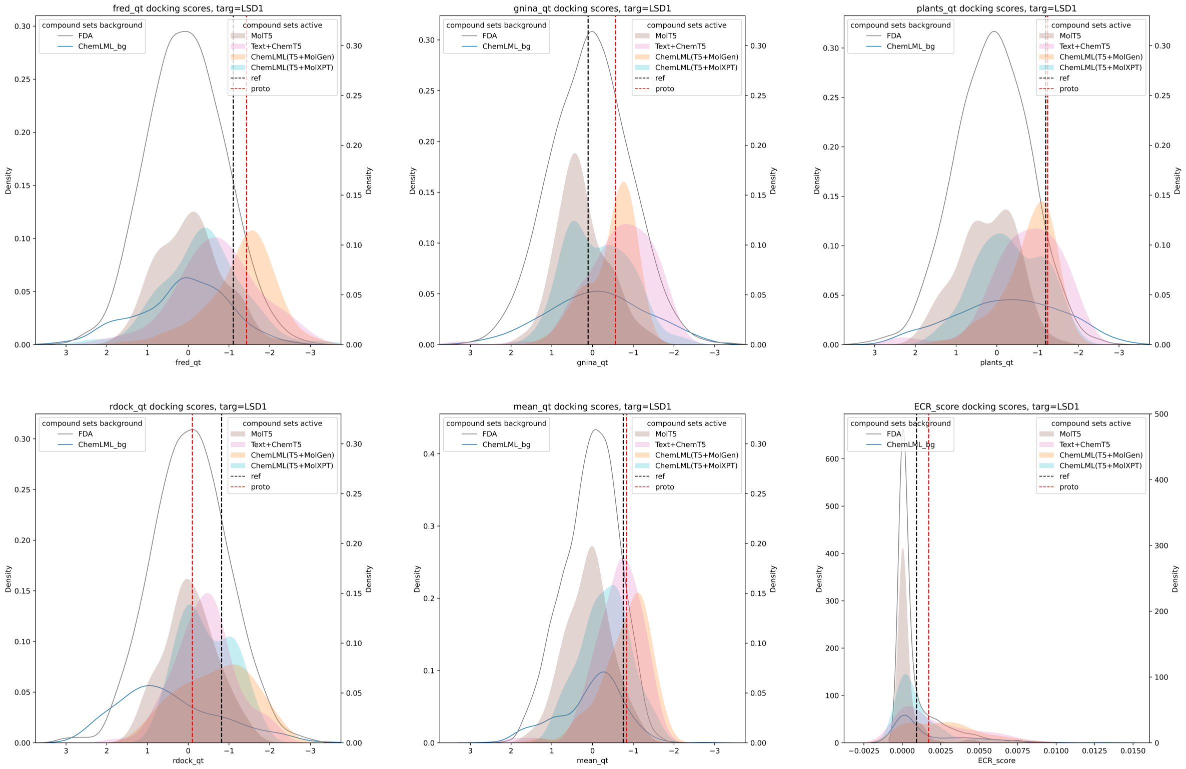

Figure S6: Individual docking program scores for LSD1.

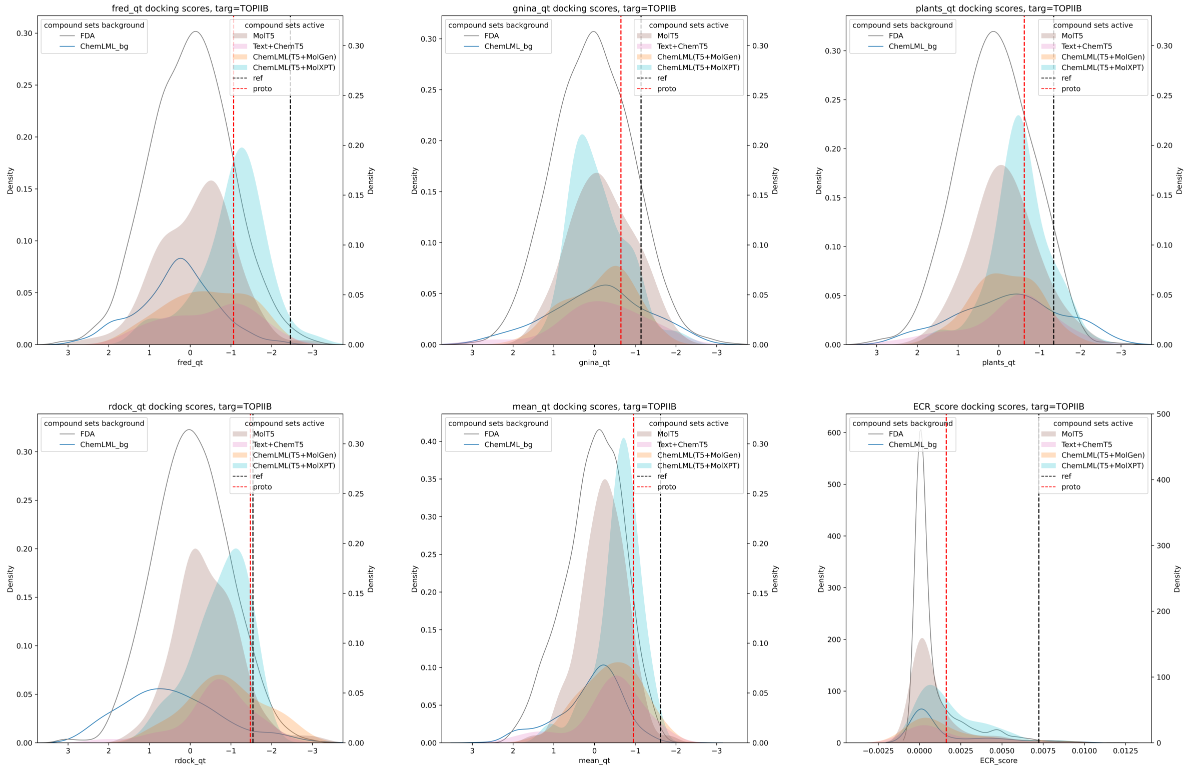

Figure S7: Individual docking program scores for TOPIIB.

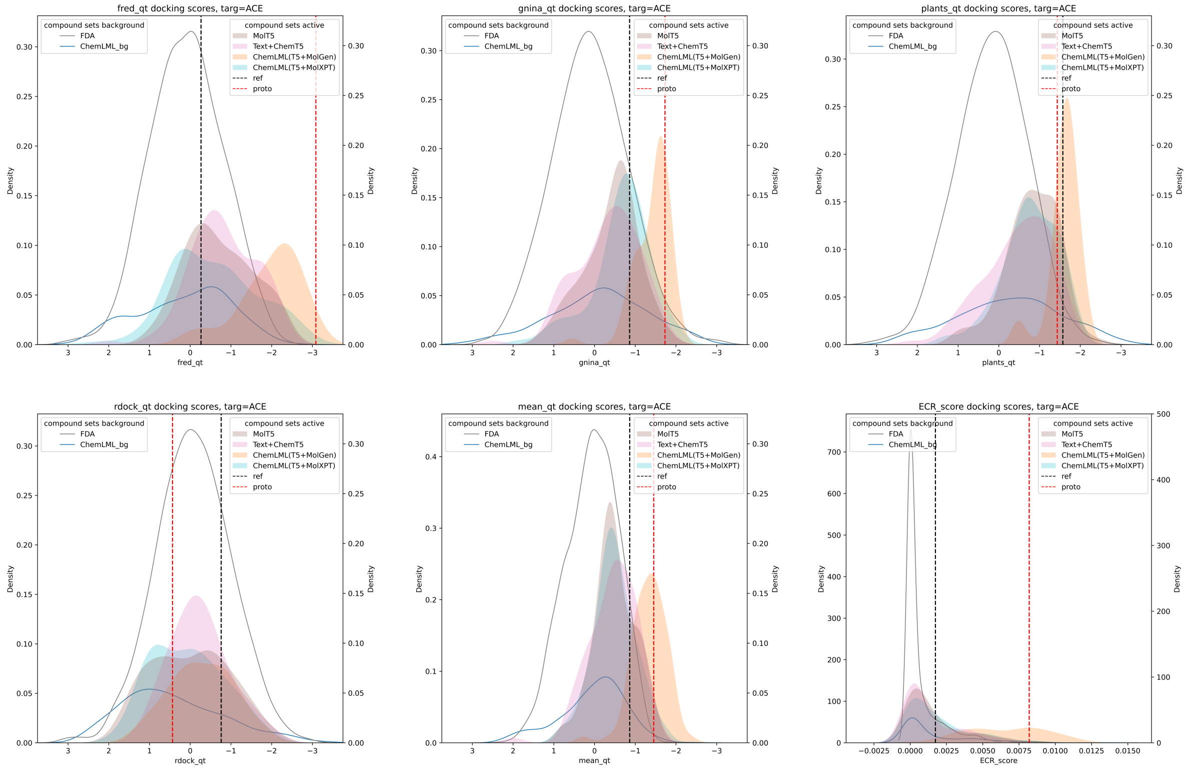

Figure S8: Individual docking program scores for ACE.

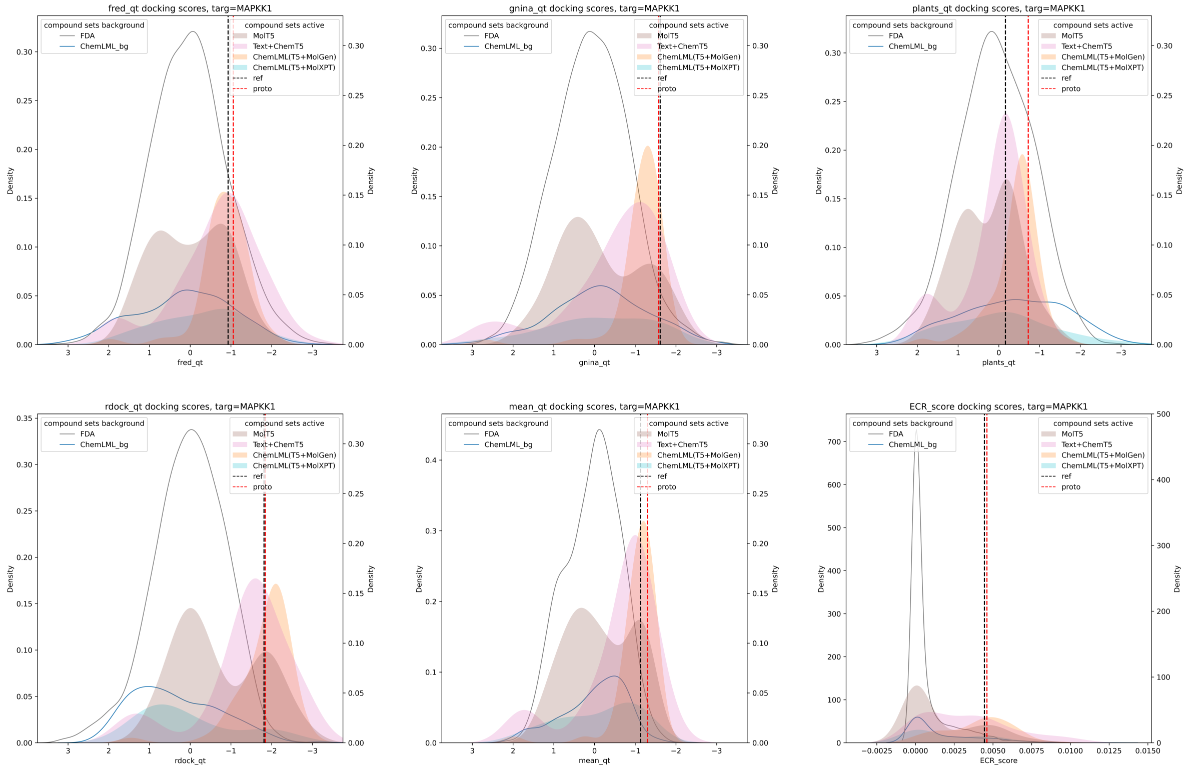

Figure S9: Individual docking program scores for MAPKK1.

Supplementary Tables

Table S1: Results of molecule generation on the PubChem-unfiltered test set.

	Models	Trainable/Total Params	Exact ↑	MACCS FTS ↑	RDK FTS ↑	Morgan FTS ↑	Validity ↑
Baseline methods	MolT5	248M/248M	0.031	0.475	0.347	0.278	0.555
	Text+ChemT5	223M/223M	0.022	0.486	0.355	0.253	0.674
ChemLML	T5 encoder+MolXPT	4.7M/464M	**0.031**	**0.474**	**0.347**	**0.224**	0.958
	T5 encoder+MolGen 7B	56.7M/6.64B	0.028	0.446	0.302	0.206	0.992
	SciBERT+MolGen	4.7M/317M	0.012	0.380	0.251	0.131	0.971
	Galactica 125M+MolGen	4.7M/333M	0.026	0.414	0.274	0.177	0.990
	T5 encoder+MolGen	4.7M/317M	0.012	0.408	0.260	0.162	**0.995**
	Galactica 1.3B+MolGen	7.4M/1.53B	0.025	0.410	0.269	0.182	0.994
	Galactica 6.7B+MolGen	11.5M/6.87B	0.024	0.423	0.270	0.189	0.992
	T5 encoder finetune+MolXPT	114M/464M	**0.034**	0.453	0.314	**0.229**	0.988
	SciBERT finetune+MolGen	115M/317M	0.027	0.426	0.289	0.200	0.990
	T5 encoder finetune+MolGen	114M/317M	0.029	0.458	0.325	0.208	0.991
	Galactica 125M finetune+MolGen	130M/333M	0.021	0.413	0.271	0.175	0.991
	T5 encoder finetune+MolGen 7B	166M/6.64B	0.030	**0.474**	**0.337**	0.218	**0.995**

Table S2: Text descriptions, ground truth molecules, generated molecules, and target proteins selected for the docking case study. The molecule columns also visualize the 2D structures.

Similarity	Ground truth molecule (SMILES)	Ground truth molecule (PubChem CID)	Generated molecule (SMILES)	Description	Target protein (UniProt)	Target protein structure (PDB)
Low	C/C=C1\\[C@H]2C=C(C)C[C@]1(N)c1ccc(=O)[nH]c1C2<br>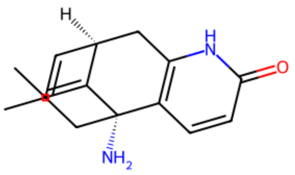	854026	CC1=CC[C@@H](CCC[C@@H](N)C=C2Cc3cccc(c3)N2)[C@@H]1N<br>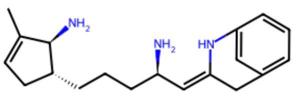	The molecule is a sesquiterpene alkaloid isolated from a club moss Huperzia serrata that has been shown to exhibit neuroprotective activity. It is also an effective inhibitor of acetylcholinesterase and has attracted interest as a therapeutic candidate for Alzheimer's disease. It has a role as an EC 3.1.1.7 (acetylcholinesterase) inhibitor, a neuroprotective agent, a plant metabolite and a nootropic agent. It is a sesquiterpene alkaloid, a pyridone, a primary amino compound and an organic heterotricyclic compound. It is a conjugate base of a huperzine A(1+).	P22303	4EY5[74]
Medium	NC(=O)c1ncn([C@@H]2O[C@H](COP(=O)(O)O)[C@@H](O)[C@H]2O)n1<br>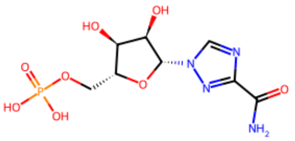	100252	NC(=O)c1cncc[n+]1N=C1N[C@H](COP(=O)(O)O)[C@@H](O)[C@H]1O<br>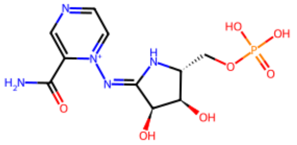	The molecule is a 1-ribosyltriazole that is ribavirin in which the hydroxy group at the 5'-position is replaced by a phosphonooxy group. It is the active metabolite of the antiviral agent ribavirin. It has a role as a human blood serum metabolite, a drug metabolite, an antiviral agent and an EC 1.1.1.205 (IMP dehydrogenase) inhibitor. It is a 1-ribosyltriazole, a ribose monophosphate, an aromatic amide, a monocarboxylic acid amide and a primary carboxamide. It is functionally related to a ribavirin. It is a conjugate acid of a ribavirin 5'-monophosphate(2-).	P50097	1ME7[75]
High	CO[C@H]1/C=C\\C=C(/C)C(=O)NC2=CC(=O)C(NCCN(C)C)=C(C[C@@H](C)C[C@H](OC)[C@H](O)[C@@H](C)/C=C(\\C)[C@@H]1OC(N)=O)C2=O<br>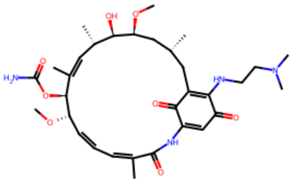	5288674	C=C[CH]C[C@@H]1CC(=O)C=C(C=O)C=CC(=O)NC(=O)/C(C)=C/C=C\\[C@H](OC)[C@@H](OC(N)=O)/C(C)=C/[C@H](C)[C@@H](O)[C@@H](OC)C1<br>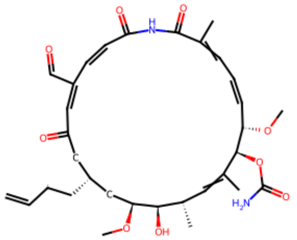	The molecule is a 19-membered macrocyle that is geldanamycin in which the methoxy group attached to the benzoquinone moiety has been replaced by a 2-(N,N-dimethylamino)ethylamino group. It has a role as a Hsp90 inhibitor. It is a secondary amino compound, a tertiary amino compound, an ansamycin, a member of 1,4-benzoquinones and a carbamate ester. It is functionally related to a geldanamycin.	P07900	1OSF[76]

Similarity	Ground truth molecule (SMILES)	Ground truth molecule (PubChem CID)	Generated molecule (SMILES)	Description	Target protein (UniProt)	Target protein structure (PDB)
High	CCOC(=O)[C@H](CCc1ccccc1)N[C@@H](C)C(=O)N1CCC[C@H]1C(=O)O.O=C(O)/C=C\\C(=O)O<br>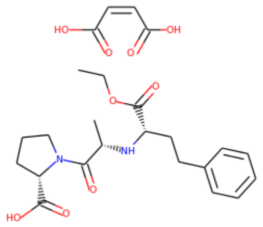	5388961	C[C@H](N[C@@H](CCc1ccccc1)C(=O)O)C(=O)N1CCC[C@H]1C(=O)OOOC(Cc1ccccc1)C(=O)O<br>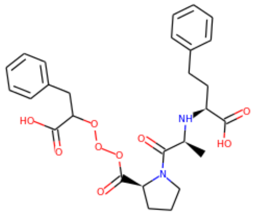	The molecule is the maleic acid salt of enalapril. It contains one molecule of maleic acid for each molecule of enalapril. Following oral administration, the ethyl ester group of enalapril is hydrolysed to afford the corresponding carboxylic acid, enalaprilat, an angiotensin-converting enzyme (ACE) inhibitor. Enalapril is thus a prodrug for enalaprilat (which, unlike enalapril, is not absorbed by mouth), and its maleate is used in the treatment of hypertension and heart failure, for reduction of proteinuria and renal disease in patients with nephropathies, and for the prevention of stroke, myocardial infarction, and cardiac death in high-risk patients. It has a role as an EC 3.4.15.1 (peptidyl-dipeptidase A) inhibitor, an antihypertensive agent and a prodrug. It contains an enalapril.	P12821	1UZE[77]
High	O=C1OC[C@H](Cc2ccc3c(c2)OCO3)/C1=C\\c1ccc2c(c1)OCO2<br>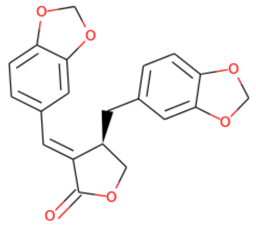	5281867	O=C1OC[C@H](Cc2ccc3c(c2)OCO3)[C@H]1Cc1ccc2c(c1)OCO2<br>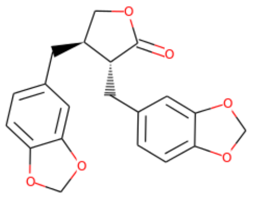	The molecule is a lignan that is dihydrofuran-2(3H)-one (gamma-butyrolactone) substituted by a 1,3-benzodioxol-5-ylmethylidene group at position 3 and a 1,3-benzodioxol-5-ylmethyl group at position 4 (the 3E,4R-isomer). It exhibits antiviral activity against SARS-CoV-2. It has a role as a T-cell proliferation inhibitor, an anti-inflammatory agent, a plant metabolite, an EC 3.4.22.69 (SARS coronavirus main proteinase) inhibitor and an anticoronaviral agent. It is a member of benzodioxoles, a lignan and a gamma-lactone.	P0C6X7	1UK4[78]
High	CCO.N#CC(=C(/N)Sc1ccccc1N)/C(C#N)=C(\\N)Sc1ccccc1N<br>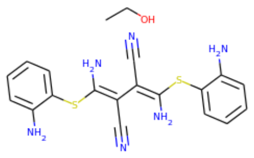	16220066	CCON=CC(=C(/N)Sc1ccccc1N)/C(C#N)=C(\\N)Sc1ccccc1N<br>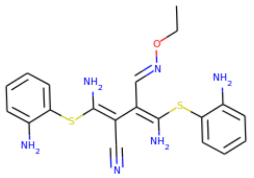	The molecule is an addition compound obtained by combining equimolar amounts of (2Z,3Z)-bisamino[(2-aminophenyl)sulfanyl]methylidenebutanedinitrile (U0126) and ethanol. An inhibitor of mitogen-activated protein kinase that also exhibits anti-cancer properties. It has a role as an EC 2.7.11.24 (mitogen-activated protein kinase) inhibitor, an apoptosis inducer, an antineoplastic agent, an antioxidant, an osteogenesis regulator and a vasoconstrictor agent. It contains an U0126.	Q02750	3EQH[79]

Similarity	Ground truth molecule (SMILES)	Ground truth molecule (PubChem CID)	Generated molecule (SMILES)	Description	Target protein (UniProt)	Target protein structure (PDB)
Low	CC(O)C(=O)O.COc1cc(NS(C)(=O)=O)ccc1Nc1c2ccccc2nc2ccccc12 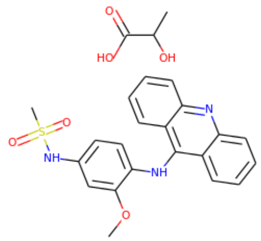	88124	NC=CC=CC1=CCc2cc(N)cc3c2c(=O)n1c1ccccc31 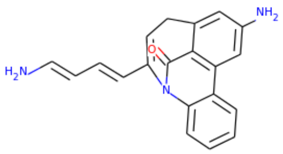	The molecule is the lactate form of amsacrine, an aminoacridine analog and topoisomerase II inhibitor, with antineoplastic activity. Although the exact relationship between DNA binding and its activity has yet to be fully elucidated, amsacrine intercalates DNA through its acridine moiety, and its nonintercalative headgroup impedes topoisomerase II activity, augmenting enzyme-mediated DNA cleavage and resulting in DNA double-strand breaks. This ultimately induces programmed cell death.	Q02880	4G0U[80]
Medium	O=C(O)c1ccc(CN2CCC(CN[C@@H]3C[C@H]3c3ccccc3)CC2)cc1 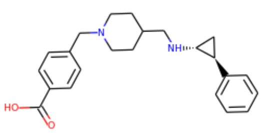	66571643	O=C(O)c1ccc(CNCCC2CCC2(NCCC2CC2)c2ccccc2)cc1 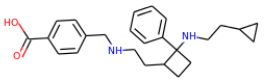	The molecule is a member of the class of piperidines that is piperidine substituted by (4-carboxyphenyl)methyl and [(1R,2S)-2-phenylcyclopropyl]aminomethyl groups at positions 1 and 4, respectively. It is a potent and irreversible inhibitor of lysine specific demethylase 1 (LSD1, also known as KDM1A). It was under clinical investigation for the treatment of acute myeloid leukaemia and small cell lung carcinoma. It has a role as an EC 1.14.99.66 (lysine-specific histone demethylase 1A) inhibitor and an antineoplastic agent. It is a member of benzoic acids, a monocarboxylic acid, a member of piperidines, a member of cyclopropanes, a tertiary amino compound, a secondary amino compound and a member of benzenes.	O60341	6NQU[81]

Molecule set	Stage	Counts (N)							
		HSP90AA1	ACE1	M^{pro}	MAPKK 1	IMPDH	LSD1	AChE	TOPIIB
FDA	docking	2956	2956	2956	2956	2954	2956	2952	2956
	omega	2977							
	download	3105							
ChemLML background	docking	657	657	657	657	657	657	657	657
	omega	663							
	generation	1000							
ChemLML(T5+MolGen)	docking	16	66	43	54	42	75	52	44
	omega	16	66	43	54	42	75	52	45
	generation	100	100	100	100	100	100	100	100
ChemLML(T5+MolXPT)	docking	99	92	95	28	99	95	92	90
	omega	99	92	95	28	99	95	92	90
	generation	100	100	100	28	100	100	100	100
MolT5	docking	97	97	98	89	98	99	96	97
	omega	97	97	98	89	99	99	96	97
	generation	100	99	100	91	100	99	100	100
Text+ChemT5	docking	83	98	94	94	96	97	86	31
	omega	83	98	94	94	99	98	86	31
	generation	100	100	100	100	100	100	100	34

Table S3: Attrition of model-generated SMILES for each target from generation to docking output. The values indicate the number of SMILES successfully generated (RDKit-canonicalized); OpenEye (OE) canonicalized, protonated, and embedded into 3D coordinates by Omega2; and ultimately docked.

Supplementary Methods

PubChem dataset

Generic PubChem descriptions may be reasonable for learning general molecule properties but violate our assumptions for evaluating text-based molecule generation, which leverages the molecular similarity principle and assumes the generated molecule should be structurally similar to the ground truth molecule. The biggest problem with PubChem-unfiltered is that many molecule descriptions are general. For example, the most frequent description, occurring 5,753 times is "The molecule is a peptide", which is uninformative. The top 10 most frequent descriptions are shown in Figure S1. Also, there are descriptions like "The molecule is a natural product found in" when there can be hundreds of natural products produced by a single species. For instance, the fungal genera *Aspergillus* and *Penicillium* are associated with 3,091 and 2,550 natural products, respectively, and the bacterial genus *Streptomyces* is associated with 5,755 natural products[82].

In addition, there are counterintuitive descriptions. One PubChem-unfiltered example has the description "The molecule is a mineral" and the SMILES of oxygen. It turns out that "minerals" is indeed in the PubChem page of oxygen (CID 977). This is because "orange" $^{18}O_2$ was crystallized under special experimental conditions[83]. Thus, oxygen is recorded in the American Mineralogist Crystal Structure Database, which is cross-referenced from PubChem. In order to obtain a more meaningful dataset, we filter out descriptions less than 30 words and containing the description "natural product". This eliminates many, but not all, of the issues described above.

Even the PubChem-filtered dataset contains other potential problems. A form of data leakage arguably occurs when a synonym of the molecule is in the text description. Our dataset review noted many drug names such as loperamide, tretinoin, lanreotide, ambrisentan, nesbuvir, danusertib, and others in the descriptions. Other descriptions state that the molecule is an enantiomer of another. Yet other descriptions retain leftover IUPAC names,

which can be used to generate molecules[84]. In all of these cases, the text descriptions gives more information than expected about the chemical structure, which may inflate similarity-based evaluation metrics.

We constructed the PubChem-filtered dataset independently from the ChEBI-20 dataset. However, even though we transformed all the molecules into canonical form and made sure there were no overlapping canonical SMILES between the PubChem-filtered and the ChEBI-20 datasets, there were still molecules that have 100% similarity between PubChem-filtered and ChEBI-20. We did not exhaustively remove these molecules in the datasets. Thus, we cannot guarantee that PubChem-filtered is entirely disjoint from the ChEBI-20 training and validation splits.

In the evaluations, we directly test MolT5 and Text+ChemT5's performance on the test set without further finetuning. For fair comparison, we also train ChemLML models on ChEBI-20 and test on the PubChem test set without finetuning. To make sure the PubChem test set corpus aligns with the ChEBI-20 training corpus, we manually replace each molecule's IUPAC name with "This molecule" and delete "with data available" at the end of the sentence.

Note that the typo "macrocyle" in the high similarity description in Table S2 appears in the original PubChem data and was not introduced by our processing.

Evaluation metrics

Fréchet ChemNet Distance[85] is a common evaluation metric for generative molecular models. It uses the embedding of the molecules in ChemNet[86], a long short-term memory-based model, to detect whether generated molecules are diverse and have similar properties as real molecules. We do not use this metric because our preliminary results and an independent evaluation[87] found it is highly sensitive to the sample size and molecule padding length.

Previous work in molecule generation also used metrics such as diversity and novelty for evaluating molecule generation. However, these metrics primarily focus on sampling the

chemical space to generate diverse molecules, which are not well-suited for description-guided molecule design.

Levenshtein distance and BiLingual Evaluation Understudy (BLEU) scores have also been used previously to compare string representations of molecules. However, these two scores that are common in NLP are not as suitable for molecules. Imagine the case where the generated molecule exactly matches the ground truth molecule at every character except the last right parenthesis, which makes it fail to close the aromatic ring. It will a yield high BLEU score and low Levenshtein distance, despite the generated molecule being chemically invalid.

Text+ChemT5 tasks

The Text+ChemT5[12] tasks are:

- mol2mol: This task contains forward reaction and retrosynthesis subtasks. In the forward reaction task, given reagents and/or enzymes, the model needs to generate the main product of the chemical reaction. For retrosynthesis, given the product of a chemical reaction, the model needs to find the reagents and/or enzymes.

- mol2text: Given the molecule represented as SMILES, the model needs to generate a textual description of the molecule.

- text2mol: Given the textual description of a molecule, the model needs to generate the SMILES representation the molecule.

- text2text: Given the natural language description of a chemical reaction, the model needs to generates a step-wise execution protocol to carry out the reaction.

Docking case study

To select targets from each similarity bin (low, medium, and high), we manually reviewed the instances in each bin. We selected the first five descriptions that pertained to a single, specific

protein target as opposed to multiple targets or inhibition of a biological process. Then, we examined the ground truth molecules in PubChem. We preferred experimental ligand-bound structures cross-referenced from PubChem with unambiguous binding sites for search space specification in docking. In some cases, there was not a suitable ligand-bound structure directly cross-referenced from PubChem, but we were able to find a suitable structure in the RCSB Protein Data Bank[88]. We used DrugBank[89] to confirm protein targets of the ground truth molecules as needed. Table S2 provides more information about the ground truth molecules and target proteins.

For each target protein, we docked the ground truth molecule, generated molecules, and control molecules from two types of background distributions. The first background distribution originally contained 3,082 small molecules (3,105 total substances) from the Selleck L1300 FDA-approved Library (downloaded 2024-03-25). The second background distribution originally contained 1,000 generated molecules from the ChemLML(T5+MolGen) model sampling text descriptions from PubChem-filtered. Due to molecule pre-processing, docking-based scoring was ultimately achieved on 2,956 and 657 compounds from these background sets, respectively (Table S3). Additional components within generated SMILES, like complexed waters or counterions on salts, were stripped from parent molecular species prior to RDKit SMILES canonicalization. Using OpenEye applications (Cadence Molecular Sciences, Santa Fe, NM), small molecule sets were processed from SMILES inputs into 3D conformers using Omega2-v5.0.0.3 and assigned partial charges (MMFF) using MolCharge from QUACPAC v2.2.3.3[90]. The most common error in 3D conformer generation involved missing force field parameters. The greatest attrition occurred due to the challenge of building macrocycles from generated SMILES for the HSP90AA1 target. Despite running Omega2 in "macrocycle" mode, only 16 of 100 syntactically valid SMILES (mostly macrocycles) generated by ChemLML(T5+MolGen) for this target produced dockable structures.

Protein target structures for docking were downloaded from RCSB.org[88]: HSP90AA1 (PDB: 1OSF), IMPDH (1ME7), AChE (4EY5), ACE (1UZE), M^{pro} (1UK4), MAPKK1

(3EQH), LSD1 (6TE1), and TOPIIB (4G0U) in PDB format and processed using the DockPrep utility in ChimeraX v1.7.1[91]. Compounds were docked with four different docking programs: FRED 4.3.0.3 (Cadence Molecular Sciences), Gnina v1.1 (`https://github.com/gnina/gnina`), PLANTS v1.2 (`https://github.com/discoverdata/parallel-PLANTS`), and rDock v24.03.192 (`https://github.com/CBDD/rDock`).

Docking site locations on each target were specified based on the position of the co-crystallized ground truth molecule in each target's protein crystal structure. Docking scores for each molecule, for each of the four programs, were integrated for a consensus score based on the method of exponential consensus ranking as described in Palacio-Rodríguez et al.[92].

Supplementary Results

Docking case study generated molecules

The descriptions of the IMPDH and HSP90AA1 inhibitor examples include the chemicals' functional roles as protein inhibitors as well as specific chemical structural attributes. We examined the first generated molecules generated with temperature 1 and random seed 42, shown in Table S2, to assess whether these structural attributes were present. The structure of the ground truth IMPDH inhibitor is in part described as "ribavirin in which the hydroxy group at the 5'-position is replaced by a phosphonooxy group". The corresponding generated molecule does contain the phosphonooxy (phosphate) group, but the 1,4-anhydroerythritol substructure in the ground truth molecule has an O replaced with NH in the generated molecule. In the HSP90AA1 inhibitor example, ChemLML generates a macrocycle, but it is a 22-memberered macrocycle instead of a 19-membered macrocycle. The generated molecule lacks the 1,4-benzoquinone substructure from the description but does contain the carbamate ester substructure. Even in the AChE inhibitor example, where specific details on structure are absent in the description text, key unsaturated bicyclo(3.3.1)nonyl substructures are present in both the ground truth and generated compounds. In these limited examples, ChemLML retains some structural properties in the generated molecules but omits others. Furthermore, when we cluster the molecules that ChemLML generates for each target, we observe that they reflect diverse chemical structures.
